# After the storm—Perspectives on the taxonomy of *Lactobacillaceae*

**DOI:** 10.3168/jdsc.2021-0183

**Published:** 2022-03-31

**Authors:** Nanzhen Qiao, Stijn Wittouck, Paola Mattarelli, Jinshui Zheng, Sarah Lebeer, Giovanna E. Felis, Michael G. Gänzle

**Affiliations:** 1 Department of Agricultural, Food and Nutritional Science, University of Alberta, Edmonton, Canada T6G 2P5; 2 Department of Bioscience Engineering, Research Group Environmental Ecology and Applied Microbiology, University of Antwerp, Groenenborgerlaan 171, 2020 Antwerp, Belgium; 3 Department of Agricultural and Food Sciences, University of Bologna, Viale Fanin 42, 40127 Bologna, Italy; 4 State Key Laboratory of Agricultural Microbiology, Huazhong Agricultural University, Wuhan, 430070, China; 5 Department of Biotechnology, Verona University Culture Collection, University of Verona, Strada le Grazie, 15, 37134 Verona, Italy

## Abstract

In 2020, a taxonomic reorganization of the lactic acid bacteria reclassified over 300 species in 7 genera and 2 families into one family, the *Lactobacillaceae*, with 31 genera including 23 new genera to include organisms formerly classified as *Lactobacillus* species. This communication aims to provide a debrief on the taxonomic reorganization of lactobacilli to identify shortcomings in the proposed taxonomic framework, and to outline perspectives and opportunities provided by the current taxonomy of the *Lactobacillaceae*. The current taxonomy of lactobacilli not only necessitates becoming familiar with 23 new genus names but also provides substantial new opportunities in scientific discovery and regulatory approval of these organisms. First, description of new species in the *Lactobacillaceae* is facilitated and a solid framework for description of novel genera is provided. Second, the current taxonomy greatly enhances the resolution of genus-level sequencing approaches (e.g., 16S rRNA–based metagenomics) when identifying the composition and function of microbial communities. Third, the current taxonomy greatly facilitates the formulation of hypotheses linking phylogeny to metabolism and ecology of lactobacilli.

## Highlights

• The taxonomy of *Lactobacillaceae* was modified substantially in 2020.
• The current taxonomy facilitates the description of new species and genera.
• The current taxonomy enhances the resolution of genus-level sequencing approaches.
• Studies linking phylogeny to metabolism and ecology of lactobacilli are facilitated.

## Graphical Abstract

**Figure:**
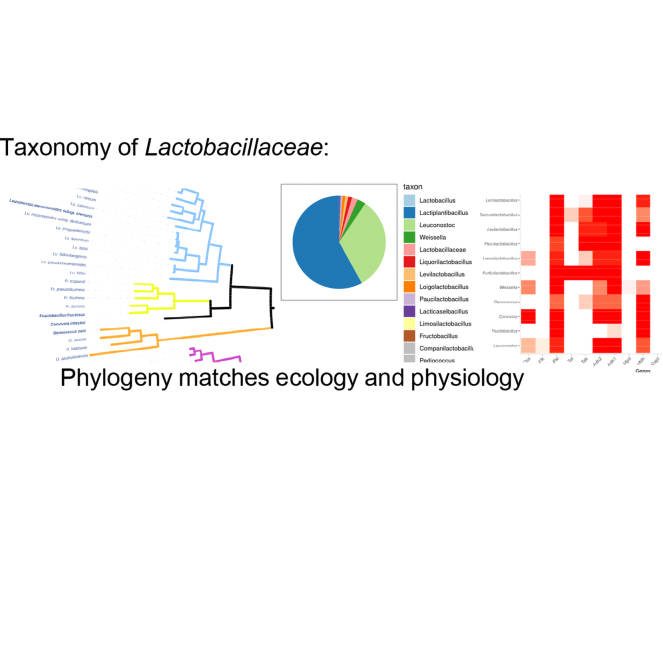
**Summary:** This communication aims to provide a “debrief” on the taxonomic reorganization of lactobacilli in 2020 and to outline perspectives and opportunities that are provided by the current taxonomy of the *Lactobacillaceae*. The new taxonomy of lactobacilli not only comes with the need to become familiar with 23 new genus names but also provides substantial new opportunities in scientific discovery and regulatory approval of these organisms. First, the description of new species in the *Lactobacillaceae* is facilitated and a solid framework for description of novel genera is provided. Second, the current taxonomy greatly enhances the resolution of genus-level sequencing approaches (e.g., 16S rRNA—based metagenomics) when identifying composition and function of microbial communities. Third, the current taxonomy greatly facilitates the formulation of hypotheses linking phylogeny to metabolism and ecology of lactobacilli.

Members of the *Lactobacillaceae* are of major importance to human health, well-being, and economic activities because they occur in most food and feed fermentations (Gänzle, 2015) and constitute important members of intestinal microbiota in many animals, including farm animals (Walter, 2008). Food-fermenting *Lactobacillaceae* have a safe tradition of food use (Bourdichon et al., 2019) and were awarded qualified presumption of safety (QPS) or generally regarded as safe (GRAS) status by the European Food Safety Authority and the US Food and Drug Administration, respectively (Koutsoumanis et al., 2020). Several species of the *Lactobacillaceae* are also used as probiotics to improve human or animal health (Hill et al., 2014).

In 2020, a taxonomic reorganization of the lactic acid bacteria reclassified over 300 species in 7 genera and 2 families into one family, the *Lactobacillaceae*, with 31 genera including *Lactobacillus*, *Paralactobacillus*, *Pediococcus*, *Weissella*, *Fructobacillus*, *Convivina*, *Oenococcus*, *Leuconostoc*, and 23 new genera that comprise organisms formerly classified as *Lactobacillus* species (Zheng et al., 2020). Sequences of 16S rRNA genes are insufficient to disentangle the phylogenetic relationships of lactobacilli (Holzapfel and Wood, 2014), and the task could thus not be undertaken until genome sequences of most type strains of lactobacilli became available (Zheng et al., 2015). This communication aims to provide a “debrief” on the taxonomic reorganization of lactobacilli to outline perspectives and opportunities provided by the current taxonomy of the *Lactobacillaceae*.

The taxonomic framework for the lactobacilli was finalized late in 2019 and refers to all 261 species of lactobacilli that were described in March 2020 (Zheng et al., 2020). Since 2020, 37 new species have been described (Figure 1). All of these species match the ecological and metabolic traits of the respective genera that were proposed earlier (Zheng et al., 2020). With few exceptions, new *Lactobacillus* and *Limosilactobacillus* species were described with isolates from vertebrate-adapted animal habitats; new *Bombilactobacillus* and *Apilactobacillus* species were described with isolates from insects and species of nomadic, environmental, or plant-associated genera were described predominantly with isolates from spontaneously fermented vegetables.

**Figure 1:**
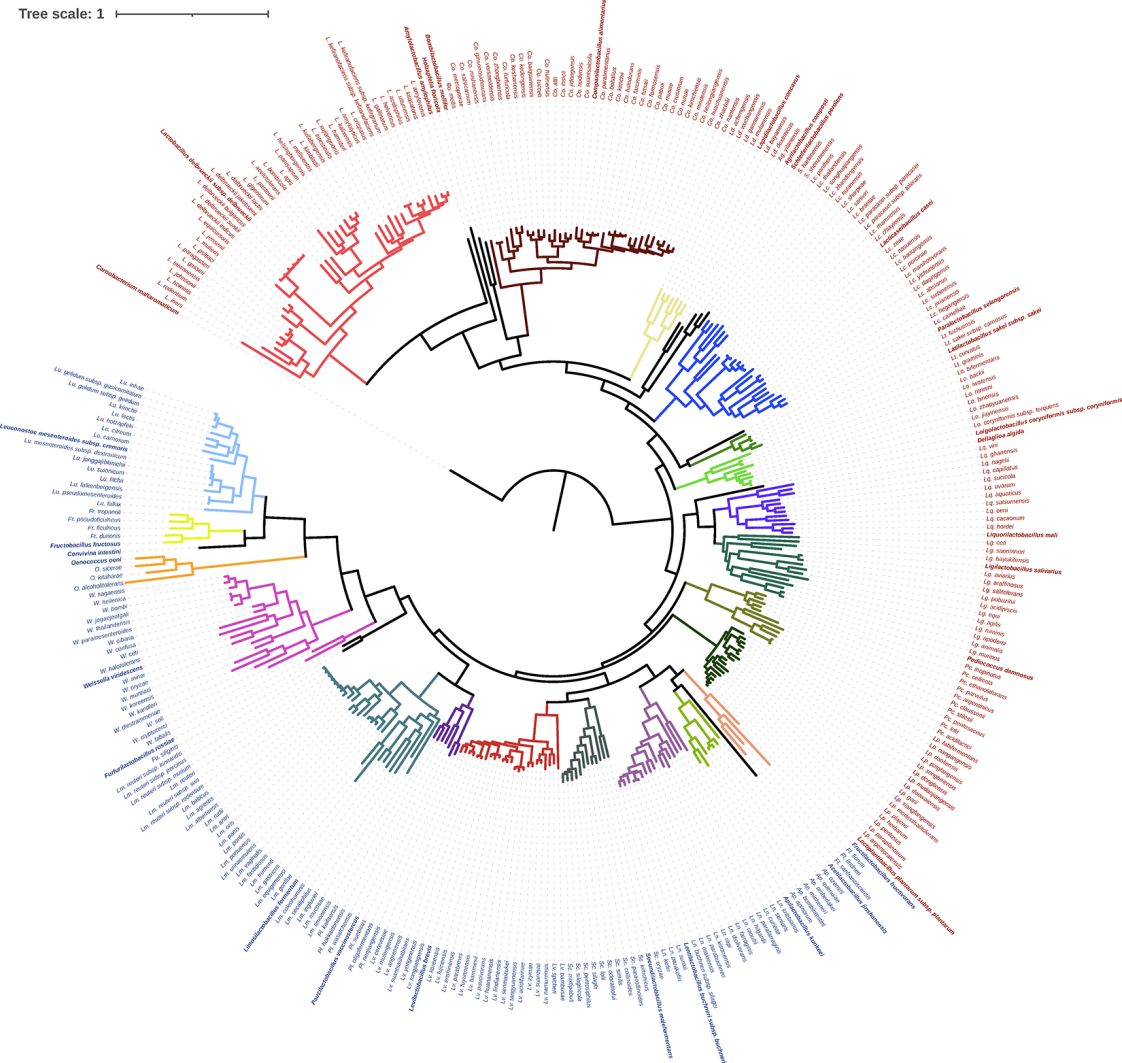
Core genome phylogenetic tree of 336 type strains in the *Lactobacillaceae* with *Carnobacterium maltaromaticum* as an outgroup. The maximum likelihood tree was analyzed on the basis of concatenated protein sequences of single-copy core genes as described in Zheng et al. (2020). Bootstrap support was calculated from 1,000 replicates; all values were >90%. Species of the same genus share the same branch color. Names of homofermentative and heterofermentative species are shown in red and blue, respectively.

The addition of more than 40 genomes since 2019 confirmed that the *Lactobacillaceae* are monophyletic only if the former *Leuconostocaceae* are included (Figure 1; Zheng et al., 2020). In addition, the node connecting the former *Leuconostocaceae* with the remaining *Lactobacillaceae* can be placed with greater confidence. Within heterofermentative organisms, the coccus- or coccoid-shaped genera form a monophyletic group that is most closely related to *Furfurilactobacillus* (Figure 1). The taxonomic framework proposed by Zheng et al. (2020) has thus been confirmed by subsequent phylogenetic analyses and new species descriptions. Updates on novel species and typical genus-level properties of lactobacilli continue to be provided at the websites www.lactobacillus.ualberta.ca and www.lactobacillus.uantwerpen.be.

Before 2020, description of new genera in the *Lactobacillaceae* was essentially impossible because it would have left the remaining genus *Lactobacillus* fragmented. The conceptual framework proposed by Zheng et al. (2020) thus facilitates the description of new taxa in the *Lactobacillaceae*. New isolates are readily identified at the genus level by 16S rRNA gene sequence analysis, as exemplified by all 37 new species in the *Lactobacillaceae* that have been described since 2020.

Currently, new species descriptions are based on an average nucleotide identity (**ANI**) value of <95% to a known type strain as the recognized threshold for delineation of new species. A pragmatic approach uses 16S rRNA gene similarity as a first step for identification. If the similarity is <98.65%, the isolate represents a novel species; if the 16S rRNA gene sequence identity is >98.65%, the ANI informs the species-level taxonomy (Chun et al., 2018). Irrespective of the methods used to derive numerical thresholds, taxonomists aim to base the description of new taxa on a combination of phenotypic and genotypic traits. Bacterial strains that have an ANI of <95 to 96% are generally differentiated by relevant ecological and metabolic properties. For example, the 6 subspecies of *Limosilactobacillus reuteri* share an ANI of <96% and are also differentiated by adaptation to different host animals and specific metabolic traits (Li et al., 2021). Similarly, the species *Lacticaseibacillus casei* and *Lacticaseibacillus paracasei* are discriminated by specific oxidative stress tolerance genes, which also support their persistence in different habitats (Wuyts et al., 2017; De Boeck et al., 2020).

Genus- or family-level taxonomy is generally achieved by comparison of protein rather than nucleotide sequences (Parks et al., 2018; Zheng et al., 2020). The taxonomic framework for reclassification of lactobacilli provided clear criteria for delineation of new genera: (1) genera are monophyletic, (2) intra- and intergenus pairwise AA identity (**AAI**)/AAI of conserved genes (**cAAI**) values show minimal overlap, and (3) genera are differentiated by characteristic metabolic or ecological traits. In the *Lactobacillaceae*, inter- and intragenus cAAI values are generally lower and higher, respectively, than 68%, with a transition zone of 65 to 71% (Zheng et al., 2020). Novel isolates can be assigned to known genera if the 16S rRNA phylogenetic tree places the isolate within a current genus. If an isolate does not consistently cluster with species of a genus or if analyses of different housekeeping genes do not provide consistent results, core genome phylogeny and AAI or cAAI values are necessary to inform whether the isolate constitutes a new genus.

The current taxonomy may also facilitate regulatory approval of *Lactobacillaceae* for use in food and feed because antibiotic resistance and formation of biogenic amines, 2 of the criteria used in safety assessment of the *Lactobacillaceae*, are partly related to the current genus-level taxonomy (Rychen et al., 2018). Recognizing the advantages of the new taxonomy, government agencies, including the US Food and Drug Administration, Health Canada, and the European Food Safety Authority, have started to use the current nomenclature.

DNA sequencing techniques have allowed the study of microbial communities on an unprecedented scale. The taxonomic resolution of sequencing approaches using 16S rRNA amplicons, however, is limited to the genus level (Johnson et al., 2019). The split of the genus *Lactobacillus* into phylogenetically and ecologically coherent genera therefore increases the resolution of this technique to gain new insights into microbial ecosystems. This is especially true for ecosystems in which lactobacilli play an important role, such as food fermentations, the human vagina, and the intestine of many animals. Most 16S rRNA sequence databases have now adopted the taxonomic changes of the *Lactobacillaceae*: SILVA from version 138.1 (Yilmaz et al., 2014), RDP from version 18 (Cole et al., 2014), GTDB from version 05-RS95 (Parks et al., 2022), and EzBioCloud from version 07/07/2021 (Yoon et al., 2017). The National Center for Biotechnology Information (NCBI) taxonomy database (https://www.ncbi.nlm.nih.gov) has been updated with the new taxonomy (Federhen, 2012) but the former names of the records containing 16S sequences are maintained, which thus still follow the old taxonomy. This means that, for example, in NCBI BLAST results, the “description” field in the results table provides the old name whereas the “scientific name” field provides the current name. The Greengenes 16S rRNA database is no longer being maintained and its most recent version still follows the old taxonomy (McDonald et al., 2012). To enable the reclassification of lactobacilli in 16S amplicon data sets without having to fully update and rerun the computational pipeline, we have developed a script and a custom database that are available at www.github.com/swittouck/reclassify_lactos.

To illustrate the increased resolution provided by the new taxonomy, we reclassified lactobacilli in samples taken from carrot juice fermentations (Wuyts et al., 2018), kefir (Walsh et al., 2016), and vaginal swabs (Ahannach et al., 2021) (Figure 2). The old taxonomy suggested that the most abundant genera in carrot fermentations are *Lactobacillus*, *Leuconostoc*, and *Weissella* (Figure 2). Use of the current taxonomy demonstrates that the majority of the lactobacilli belong to the genus *Lactiplantibacillus*, with smaller proportions belonging to the genera *Liquorilactobacillus*, *Levilactobacillus*, *Loigolactobacillus*, and *Paucilactobacillus*, thus providing a much more precise description. In kefir and vaginal samples, reanalysis of data with the current taxonomy revealed the presence of *Lentilactobacillus* and *Limosilactobacillus*, respectively, as minor components of the microbial communities (Figure 2). Few lactobacilli cannot be classified to known genera using V4 16S rRNA amplicon sequences. Sequencing of other housekeeping genes (e.g., *groEL*) improves the species-level identification of lactobacilli (Xie et al., 2019).

**Figure 2:**
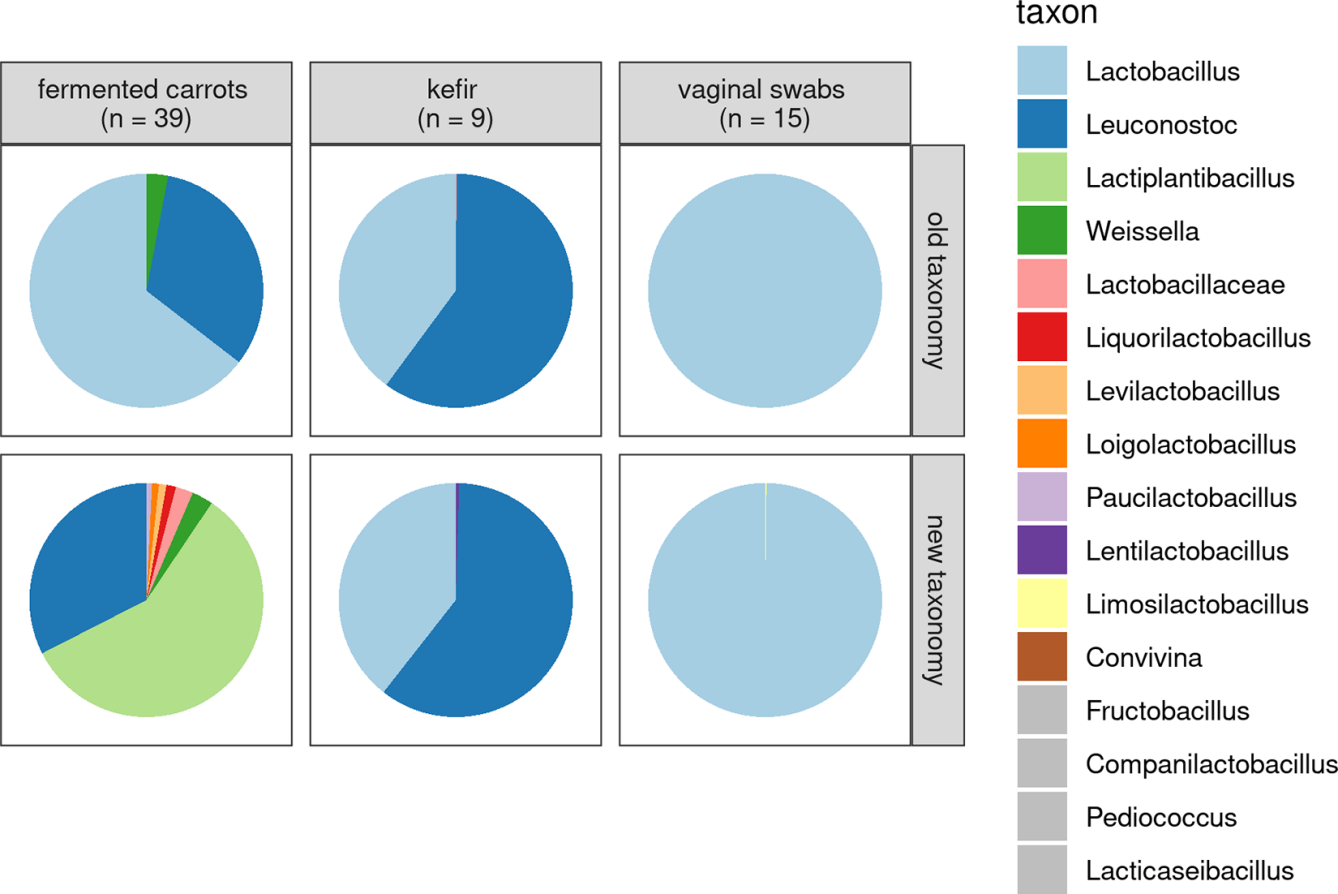
Mean abundance of *Lactobacillaceae* genera, relative to total *Lactobacillaceae* abundance, in samples from d 30 of carrot juice fermentations (left; Wuyts et al., 2018), in kefir samples (middle; Walsh et al., 2016), and in swabs from the human vagina (right; Ahannach et al., 2021).

The phylogenetic diversity of the *Lactobacillaceae* is matched by their metabolic diversity in terms of oxygen tolerance, the organic acids or carbohydrates that are utilized, and the spectrum of metabolites that are produced (Gänzle and Follador, 2012; Gänzle, 2015; Wuyts et al., 2017). Until 2020, metabolic preferences were considered a species-specific trait, which was a difficult proposition in a genus with more than 250 species. The current taxonomy facilitates the association of the taxonomy or organisms with their ecology and metabolic preferences and at 3 levels.

First, the fermentation pathways for hexoses—homofermentation or heterofermentation—are now a genus-level trait. Heterofermentative organisms form a monophyletic clade, with *Lactiplantibacillus* as the evolutionary link between homofermentative and heterofermentative species (Figure 1; Zheng et al., 2020). Heterofermentative lactic acid bacteria lack phosphofructokinase, the key enzyme of glycolysis (Figure 3), even though other carbohydrate kinases (e.g., ribokinase) are occasionally misidentified as phosphofructokinase. Homofermentative and heterofermentative *Lactobacillaceae* also differ in other major metabolic traits (Figure 3A).

**Figure 3:**
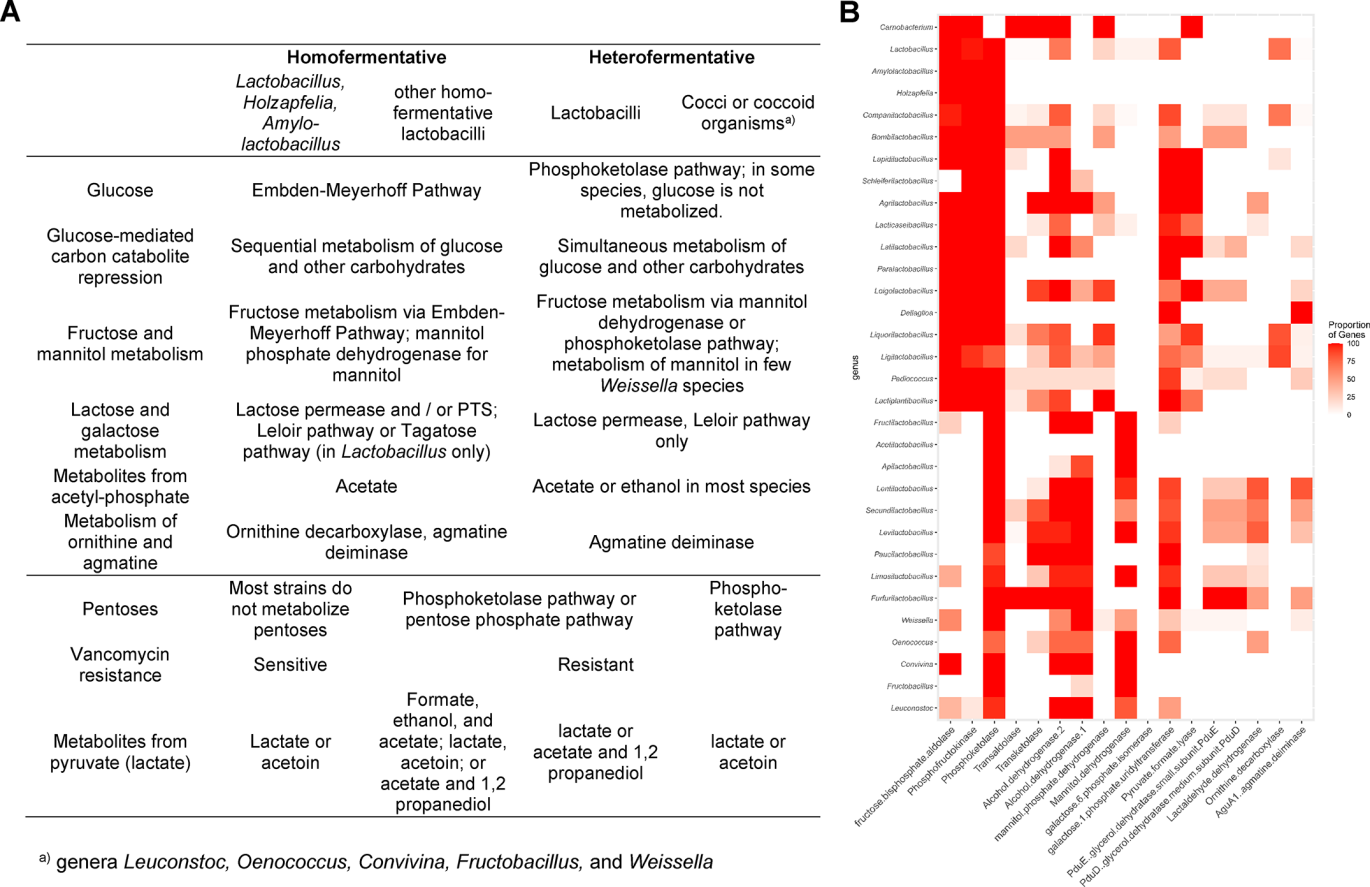
(A) Metabolic and physiological features that differentiate homofermentative and heterofermentative *Lactobacillaceae*, and the major phylogenetic groups within the homofermentative and the heterofermentative *Lactobacillaceae*. (B) In silico identification of genus-level metabolic traits of the *Lactobacillaceae.* Genes were identified in genomes of the 336 type strains in the *Lactobacillaceae* by BLASTp search (https://blast.ncbi.nlm.nih.gov/Blast.cgi?PAGE=Proteins) with 75% coverage and 50% identity as threshold values. The heatmap depicts the percentage of type species in each genus that harbor the gene, where red = present in all type strains of a genus and white = absent in all type strains.

Second, the current taxonomy facilitates the identification of genus-specific traits that were all but obscured by the sheer number of species in the genus *Lactobacillus* until 2020. For example, the insect-adapted *Bombilactobacillus* and *Apilactobacillus* species ferment only a few carbohydrates; in particular, *Apilactobacillus* species ferment only a few sugars other than fructose (Vuong and McFrederick, 2019). Other heterofermentative lactobacilli, particularly *Paucilactobacillus* species and some *Secundilactobacillus* species, specialized on habitats that are hexose-depleted, lack mannitol dehydrogenase, and ferment mainly or only pentoses (Figure 3). Several *Weissella* species differ from most other heterofermentative lactobacilli by the presence of mannitol-phosphate dehydrogenase for mannitol catabolism rather than mannitol dehydrogenase for fructose conversion (Figure 3).

Third, the current taxonomy enables identification of metabolic properties that relate to lifestyle (Duar et al., 2017) or co-evolution with other bacteria (Tannock et al., 2012; Lin et al., 2018). Few genera are adapted to vertebrate animals or to insects, respectively, and the current taxonomy thus enables identification of metabolic and physiological traits that are required for ecological fitness in these ecosystems (Vuong and McFrederick, 2019; Li and Gänzle, 2020). The link of metabolism, ecology, and phylogeny will also facilitate the identification of probiotic effector molecules that underpin the health benefits of probiotic lactobacilli (Lebeer et al., 2018).

Bacterial mutualism or cross-feeding is a key element of carbohydrate metabolism by intestinal microbiota (Louis and Flint, 2017; Cheng et al., 2020) and in other ecosystems (Oña et al., 2021). In the *Lactobacillaceae*, metabolic mutualism is mediated by proteases and glycosyl hydrolases but also by metabolites, including mannitol, lactic acid, glycerol, ornithine, and agmatine, which are further converted by other organisms (Figure 3). Mannitol, lactic acid, and ornithine are metabolites of other *Lactobacillaceae*; glycerol and agmatine are metabolites of *Saccharomyces cerevisiae* and *Enterobacteriaceae*, respectively, organisms that cohabitate with lactobacilli in plant-associated habitats (Nevoigt and Stahl, 1997; Iyer et al., 2003).

In conclusion, the current taxonomy of lactobacilli proposed by Zheng et al. (2020) provides new opportunities in scientific discovery and facilitates regulatory approval. First, the description of new species in the *Lactobacillaceae* is facilitated and a solid framework for description of novel genera is provided. Second, the current taxonomy enhances the resolution of genus-level sequencing approaches such as 16S rRNA–based metagenomics. Third, the current taxonomy enables the formulation of hypotheses linking phylogeny to metabolism and ecology of lactobacilli. In the absence of a natural system for delineation of bacterial taxa, taxonomy means no more and no less than “to give things a name.” The taxonomy of lactobacilli is thus an example on how the availability of appropriate nomenclature furthers scientific discovery.
